# Investigating the Influence of Column Depth on the Treatment of Textile Wastewater Using Natural Zeolite

**DOI:** 10.3390/molecules26227030

**Published:** 2021-11-21

**Authors:** Timoth Mkilima, Kulyash Meiramkulova, Ubaidulayeva Nurbala, Amanbek Zandybay, Mansur Khusainov, Nurgul Nurmukhanbetova, Lyazzat Tastanova, Toghan Mashan, Abdilda Meirbekov

**Affiliations:** 1 Department of Civil Engineering, Faculty of Architecture and Construction, L.N. Gumilyov Eurasian National University, Satpayev Street 2, 010000 Nur-Sultan, Kazakhstan; 2 Department of Environmental Engineering and Management, Faculty of Natural Sciences, L.N. Gumilyov Eurasian National University, Satpayev Street 2, 010000 Nur-Sultan, Kazakhstan; kuleke@gmail.com (K.M.); kamanbek_z@mail.ru (A.Z.); h-mansur@mail.ru (M.K.); 3 Department of Chemistry and Chemical Technology, K. Zhubanov Aktobe Regional University, A. Moldagulova Ave. 34, 030000 Aktobe, Kazakhstan; Nurbala-76@mail.ru (U.N.); lyazzatt@mail.ru (L.T.); 4 Department of Chemistry and Biotechnology, Sh. Ualikhanov Kokshetau University, Abai Street 76, 020000 Nur-Sultan, Kazakhstan; nn_nurgul@mail.ru; 5 Department of Chemistry, Faculty of Natural Sciences, L.N. Gumilyov Eurasian National University, Satpayev Street 2, 010000 Nur-Sultan, Kazakhstan; mashan@mail.ru; 6 Department of Enviroment and Chemistry, University of Hosa Ahmet Yesevi, 161200 Turkestan, Kazakhstan; abdilda@mail.ru

**Keywords:** natural zeolite, textile wastewater treatment, depth filtration, water quality, textile production contaminants

## Abstract

Textile industry production processes generate one of the most highly polluted wastewaters in the world. Unfortunately, the field is also challenged by the availability of relatively cheap and highly effective technologies for wastewater purification. The application of natural zeolite as a depth filter offers an alternative and potential approach for textile wastewater treatment. The performance of a depth filter treatment system can be deeply affected by the column depth and the characteristics of the wastewater to be treated. Regrettably, the information on the potential of these filter materials for the purification of textile wastewater is still scarce. Therefore, this study investigated the potential applicability of natural zeolite in terms of column depth for the treatment of textile wastewater. From the analysis results, it was observed that the filtration efficiencies were relatively low (6.1 to 13.7%) for some parameters such as total dissolved solids, electrical conductivity, chemical oxygen demand, and sodium chloride when the wastewater samples were subjected to the 0.5 m column depth. Relatively high efficiency of 82 and 93.8% was observed from color and total suspended solids, respectively, when the wastewater samples were subjected to the 0.5 m column depth. Generally, the 0.75 m column depth achieved removal efficiencies ranging from 52.3% to 97.5%, whereas the 1 m column depth achieved removal efficiencies ranging from 86.9% to 99.4%. The highest removal efficiency was achieved with a combination of total suspended solids and 1 m column depth (99.4%). In summary, the treatment approach was observed to be highly effective for the removal of total suspended solids, with a 93.8% removal efficiency when the wastewater was subjected to the 0.5 m column depth, 97.5% for 0.75 m column depth, and 99.4% for 1 m column depth. Moreover, up to 218.233 mg of color per g of the filter material was captured. The results derived in this study provide useful information towards the potential applicability of natural zeolite in the textile wastewater treatment field.

## 1. Introduction

The growing demand for textile products has led to an increase in textile industries, particularly in the developing world. The most evident impact of the textile production processes in the environment is the high consumption of water and wastewater discharge, whereby it is estimated that the processes generate approximately 115–175 kg of chemical oxygen demand (COD) per ton of finished product [1]. Generally, the wastewater from textile production processes has a variable and complex mixture of pollutants, such as organic and inorganic compounds, polymers, and color [2,3]. The phenomenon has also negatively impacted human health and the environment, in general, due to its contaminated effluents [4]. The textile processing industry is among the water-intensive industrial sectors, as most of the stages in the production line require water [5,6].

More specifically, the effluents from textile production processes are characterized by a high pH value, high concentration of suspended substances, color, chlorides, nitrates, high biological oxygen demand (BOD), COD value, and metals including manganese, sodium, lead, copper, chromium, and iron [7]. Among the key steps in the production process is the application of synthetic dyes for adding color to fibers. However, a large volume of water is required in this process, which results in the discharge of substantial amounts of dyeing effluent as wastewater [8]. Therefore, treatment of such highly polluted wastewater before discharge or any sort of reuse is of great importance to protecting human health and the environment in general.

Generally, textile industry wastewater can be purified by using physical, chemical, and biological-based treatment approaches. However, each of the treatment methodologies possesses advantages (strengths) and disadvantages (weaknesses). For instance, physical wastewater treatments, such as membrane filtration systems [9,10], are seen as highly efficient in removing most wastewater pollutants but are extremely pressure-demanding systems, making them relatively expensive. Biological or natural treatment approaches, on the other hand, are well-known for their high adaptability to a wide variety of wastewater compositions [11]; however, these systems are relatively slow processes requiring large physical areas [12]. Moreover, chemical-based treatment systems such as electrochemical processes are known to be robust, require small space, are easy to operate, and remain flexible under fluctuating wastewater composition, but are also high pressure-demanding and associated with the production of by-products [13,14,15]. In summary, it should be noted that the applicability of each of the technologies is highly limited to the capital and operational cost. High energy consumption is among the most crucial challenges in applying wastewater treatment technologies, especially in low-income regions. Filtration using natural zeolite can be a potential treatment approach that provides a relatively low-cost treatment approach in the textile wastewater treatment field.

Zeolite can be defined as an alkaline mineral that is very porous while possessing a negative charge or is a microporous, three-dimensional crystalline solid of aluminium silicate with the general chemical formula shown in Equation (1).

(1) $$Na_{2}Al_{2}Si_{2}O_{8}xH_{2}O$$

Since most toxins in wastewater, such as pesticides, heavy metals, and radiation, are positively charged [16], zeolite is pulled towards them with an almost magnetic force, sucking them up into its structure [17,18,19]. Apart from the porosity of a single zeolite particle, the media formed by zeolite have many pores that do not just capture particles between the particles, but also absorb them into their pores to capture them. As previously mentioned, the whole process is done in part by the capacity of zeolite minerals for cation exchange. The cation exchange capacity makes it possible for the zeolite media to capture positive ions such as dissolved metals, sodium, and ammonia from the water [20,21].

Moreover, due to its high pore density, zeolite is also characterized by a high effective surface area, which means it can capture a sufficient number of contaminants before backwashing is needed. This phenomenon makes the zeolite medium relatively less prone to clogging. Adsorption is another pollutant-removal mechanism in wastewater that uses zeolite [22]. The adsorption process is an active effect where particles adhere to the surface of the media instead of passively getting trapped between the particles. Zeolite media have also been observed to be more resistant to chemicals compared with some other media, with a relatively high capacity of reducing certain hardness minerals in the wastewater, thus allowing them to possess the water softening capacity [23]. The softening process with zeolite is described in Equation (2).

(2) $$\left[\begin{array}{c} Ca\\ Mg \end{array}\right]*\left[\begin{array}{c} SO_{4}\\ 2Cl\\ 2HCO_{3} \end{array}\right]+Na_{2}*Z\to Z*\left[\begin{array}{c} Ca\\ Mg \end{array}\right]*\left[\begin{array}{c} Na_{2}SO_{4}\\ 2NaCl\\ 2NaHCO_{3} \end{array}\right]$$

where *Z* represents the insoluble radical framework.

In general, depth filters are developed to create a column porous medium that traps the wastewater pollutants throughout itself, compared with membrane filters, where the particles are captured on the surface [24]. However, the performance of zeolite on wastewater treatment can be highly affected by the column depth and characteristics of the wastewater to be treated. Unfortunately, the information on how zeolite column depth would influence the performance of the system on textile wastewater is still scarce. The effect of column depth using natural zeolite as a filter material for the purification of textile wastewater has not yet been captured in previous studies.

In this study, the influence of column depth on the performance of zeolite to purify textile wastewater was investigated. Three different column depths (0.5 m, 0.75 m, and 1 m) were used to investigate the influence of column depth on the treatment efficiency of the zeolite filter media. Different statistical methods, such as percent removal efficiency, correlation matrices, and box and whisker plots, were used to analyze the data distribution and performance of the system. Despite the potential of natural zeolite to provide an alternative, relatively cheap, and effective treatment approach for textile wastewater treatment, the information on the potential applicability of these filter materials for the treatment of dye-rich wastewater is still scarce. A total of twelve (12) water quality parameters (pH, color, total suspended solids (TSS), total dissolved solids (TDS), dissolved oxygen (DO), turbidity, electrical conductivity (EC), chemical oxygen demand (COD), biochemical oxygen demand (BOD), nitrate, phosphorus, and sodium chloride) were investigated based on maximum (max) and minimum (min) arithmetic mean, median, and standard deviation (STD).

## 2. Results and Discussion

### 2.1. Wastewater Characterization

The water samples were successfully analyzed based on the samples collected from the textile industry. In the study, pH was among the parameters investigated in raw wastewater. However, it should be noted that, during the treatment processes, no adjustments to alter the natural condition of the wastewater were made. Therefore, the presented pH is based on the initial pH in the raw wastewater. Generally, the pH ranged from slightly above neutral (7.5) to highly alkaline (12.3). The phenomenon is likely linked to the fact that, in the production steps of the textile industry, caustic and other detergents of an alkaline nature are used in large quantities—a phenomenon that is also reported in the study conducted by Patel et al. [25] and Hussain et al. [26]. High pH in water tends to cause numerous negative effects, including bitter taste, water pipes and water-using appliances being covered with a hard crust from deposits [27], and the effectiveness of disinfection processes using chlorine being affected [28]. Moreover, in terms of low-pH water, metals are likely to be corroded [29].

In the raw wastewater samples, the average color concentration was 1685.92 degrees, with 4090 degrees and 231 degrees being the maximum and minimum recorded concentrations, respectively. In many cases, not only can color reduce water’s aesthetically appealing quality, thus affecting its value, but colored wastewater being discharged into a water body can also significantly affect photosynthesis activities, leading to detrimental effects on the aquatic ecosystems [30]. 

The average total suspended solids (TSS) concentration in the raw wastewater was 702.17 mg/L, while 2435 mg/L and 131 mg/L were the maximum and minimum recorded concentrations, respectively. High TSS concentration levels in wastewater can have both environmental and human health effects. More specifically, TSS may block sunlight in a water body, which may halt photosynthesis processes, affecting a plant’s life and eventually decreasing the oxygen concentration levels in water [31]. Moreover, high TSS can clog gills in a water body containing aquatic organisms such as fish, thus leading to their deaths or even reducing their growth rate [32]. 

The average concentration of total dissolved solids (TDS) in the raw wastewater was 7479.83 mg/L, while 10160 mg/L and 2680 mg/L were the maximum and minimum concentrations, respectively. High TDS concentration levels in a water body tend to change the density of the water, which also determines how easily water can flow into and out of an aquatic organism′s cells [33]. As previously mentioned when discussing the effects of TSS on a water body, high concentrations of TDS also reduce water clarity, which, in turn, affects photosynthesis activities in the water. In general, TDS is used to define the quality level of drinking water due to the fact that it represents the amounts of ions in the water. Water with high TDS means contains a high amount of ions and is often characterized by an unpleasant taste and high levels of water hardness [34]. For instance, according to the world health organization (WHO), the TDS levels in drinking water should be less than 300 mg/L. The average TDS concentration recorded in this study falls under the category of greater, at 1200 mg/L, which is unacceptable as per the WHO standards [35].

The average concentrations of dissolved oxygen (DO), turbidity, and electrical conductivity (EC) were 2.88 mg/L, 164.98 mg/L, and 8249.067, respectively. Similar to color, TSS, and TDS, the DO, turbidity, and EC are important water quality parameters that determine the usability of water. High concentration levels of these parameters can significantly affect the photosynthesis activities in water and generally impact aquatic life. The average concentrations of COD and BOD were 2133.83 mg/L and 366.67 mg/L, respectively. Moreover, the average concentrations of nitrate, phosphorus, and sodium chloride were 45.63 mg/L, 2.8 mg/L, and 9.7 mg/L, respectively. 

Excessive concentrations of turbidity can be associated with a significant reduction of a water body’s aesthetic quality, as well as affecting recreational and tourism value [36]. Also, high levels of turbidity can potentially affect the growth rate of aquatic plants in water bodies as this tends to decrease the amount of light required for proper photosynthesis [37]. Turbidity can also increase water temperature because suspended particles absorb more heat [38]. 

Electrical conductivity determines the ability of water to conduct electricity, which also acts as a measure of dissolved solids in water. On the other hand, elevated levels of COD indicate that there is a greater amount of oxidizable organic matter in water, which also significantly affects the amount of dissolved oxygen. To the opposite end, a reduction in dissolved oxygen can result in elevated anaerobic conditions in water, which also has a significant effect on aquatic life [39]. Another important parameter in textile wastewater is BOD, which is also generally used to assess the levels of water quality as determined by the degree of pollution from organic matter. More specifically, BOD represents the amount of oxygen consumed by aerobic microorganisms to oxidize organic matter in water. Similar to the effects of COD in water, water with elevated levels of BOD can also significantly reduce the amount of dissolved oxygen [40].

### 2.2. Data Distribution in Raw Wastewater

**Figure 1:**
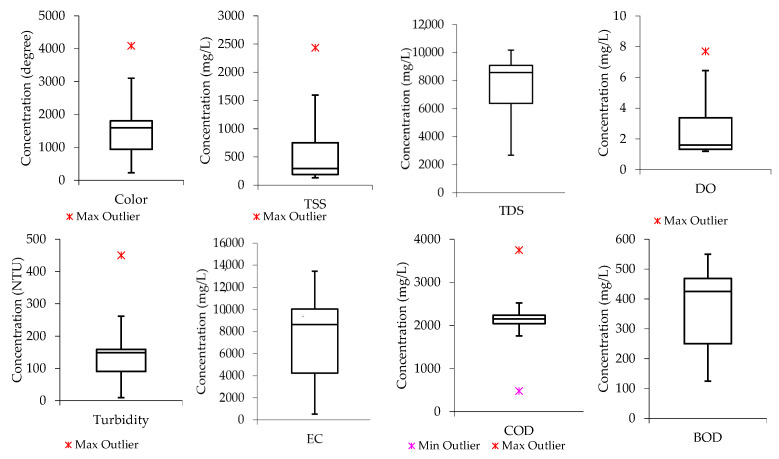
Data distribution from the raw wastewater samples for color, TSS, TDS, DO, turbidity, EC, COD, and BOD.

Boxplots were also developed to assess the nature of data distribution among the water quality parameters investigated in raw wastewater. Figure 1 indicates that the median lines for color, TDS, and BOD are closer to the upper quartile, meaning the distribution of data from the water quality parameters in the raw wastewater is negatively skewed. This shows that the data constituted a higher frequency of low concentration values than the high concentration values. For instance, the data distribution for color had most of the concertation values within the range of 1000 degrees and slightly below 2000 degrees, while TDS had most of the concentration values ranging from slightly above 6000 mg/L and below 9000 mg/L. A similar trend was observed in the literature according to the study conducted by Hajira and Abdul [41], whereby the textile wastewater from Karachi City’s industrial zone was investigated. Also, the median line for COD is closer to the middle, indicating that the COD data distribution is symmetric or normal, while that of TSS and DO are observed to be closer to the lower quartile, meaning the data constituted a higher frequency of high concentration values than low concentration values (“positively skewed”).

### 2.3. Relationship among Parameters in the Raw Wastewater

The correlation matrices were developed by first grouping the water quality parameters of interest into two groups; the grouping of the parameters was based on the potential correlation among the parameters. Group number one consisted of color, TSS, TDS, DO, and turbidity (Table 1). Group number two consisted of DO, TDS, EC, COD, and BOD (Table 4). From Table 3, it can be observed that there was a very high correlation between turbidity and color with a correlation index of 0.99, turbidity and TSS with a correlation index of 0.95, TSS and color with a correlation index of 0.94, TDS and color with a correlation index of 0.73, and DO and color (negative correlation) with a correlation index of −0.71.

Color in water is generally a result of dissolved organic material, while turbidity is a result of tiny particles suspended in the water column. The organic material in the water causes both color and turbidity, and it is why a potential correlation between the two water quality parameters always exists [42]. More specifically, the more intensive the color is in water, the more light is absorbed, and the higher the turbidity.

The high negative correlation between BOD and DO can be linked to the fact that BOD directly affects the amount of dissolved oxygen in the water, which means the greater the BOD, the more rapidly oxygen is depleted [43].

Table 2 indicates that EC is highly correlated with all the other studied parameters in this group, with a correlation index ranging from 0.86 to 0.99. The phenomenon is likely linked to the fact that EC is a measure of water’s general capability to allow the flow of electricity. To achieve this, the water has to be rich in terms of ion concentration. These conductive ions come from dissolved salts and other inorganic materials that can be expressed in terms of other water quality parameters such as TDS. Linear correlation among these parameters has been observed in the literature [44]. Furthermore, the reason that the determination of EC in water is important is that it provides a picture of how much of the dissolved substances, chemicals, and minerals are present in the water. 

It has to be noted that negative, or sometimes known as inverse, correlation is an indication that two variables move in opposite sizes and directions from one another; for instance, when one increases, the other variable decreases, and vice-versa. From Table 2, it can be observed that DO was negatively correlated with other parameters, with an indication that when the concentrations of these parameters were increasing, the DO in the raw wastewater was decreasing. The high negative correlation (−0.92) can be linked to the fact that the amount of DO used up by aerobic microorganisms to decompose the organic matter present in water is termed as BOD.

### 2.4. Treated Effluent Characterization

Table 3, Table 4 and Table 5 show that the average concentrations of the parameters decreased with column depth, except for DO, which increased. For instance, color was reduced from 1685.92 degrees in the raw wastewater to 303.75 degrees in the effluents collected from 0.5 m depth, 219.25 degrees from 0.75 m depth, and 56.90 degrees from a 1 m depth. Also, TSS was reduced from 702.17 mg/L in the raw wastewater to 43.6 mg/L in the effluents collected from 0.5 m column depth, 17.83 mg/L from 0.75 m column depth, and 4.50 mg/L from a 1 m column depth.

EC provided a contrast in that the average concentration in the raw wastewater was 7415.73 mg/L but the effluent from a 0.5 m column depth still had an average concentration of 6961.5 mg/L. A similar phenomenon can be observed with the TDS, whereby in the raw wastewater the average concentration was 7479.83 mg/L, and the average TDS concentration from 0.5 m effluent was 6458 mg/L. 

Nevertheless, the 1 m column depth was observed to be highly efficient in removing other parameters such as nitrate, phosphorous, and sodium chloride. For instance, nitrate was removed from 45.63 mg/L to 3.47 mg/L, phosphorous was removed from 2.8 mg/L to 0.26 mg/L, and sodium chloride from 9.7 mg/L to 0.68 mg/L. The study conducted by Barkouch et al. [45] on the potential effects of filter height on the removal efficiency of Cd, Cu, Pb, and Zn using slow sand filtration had similar general observations.

Despite the impressive removal performance from a 1 m depth column (Table 5), some of the parameters still do not comply with international regulations for different purposes. For example, in 1986, the US Environmental Protection Agency (EPA) established the recommended criteria for phosphorus that it should not exceed 0.1 mg/L if a stream is not discharging into reservoirs, it should not exceed 0.05 mg/L for streams discharging into reservoirs, and there must be no more than 0.024 mg/L in any reservoir [46].

### 2.5. Data Distribution in the Treated Effluent

**Figure 2:**
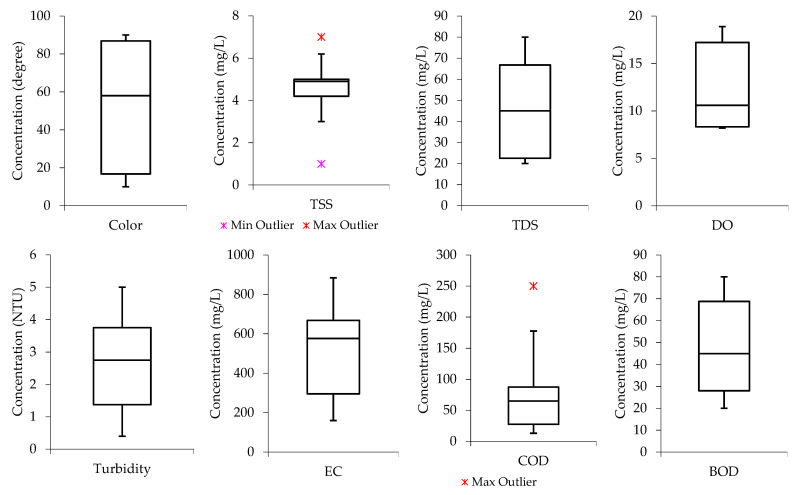
Data distribution from the 1 m column samples for color, TSS, TDS, DO, turbidity, EC, COD, and BOD.

From Figure 2, it can be observed that the color, TSS, TDS, turbidity, EC, and COD boxplot have the median lines closer to the upper quartile, indicating that the distribution of the parameters in the raw wastewater is “negatively skewed”. This means the data constituted a higher frequency of low concentration values than high concentration values. The median lines for DO and BOD are closer to the lower quartile, meaning the water quality data constituted a higher frequency of high concentration values than low concentration values (“positively skewed”). 

### 2.6. Removal Efficiencies

**Figure 3:**
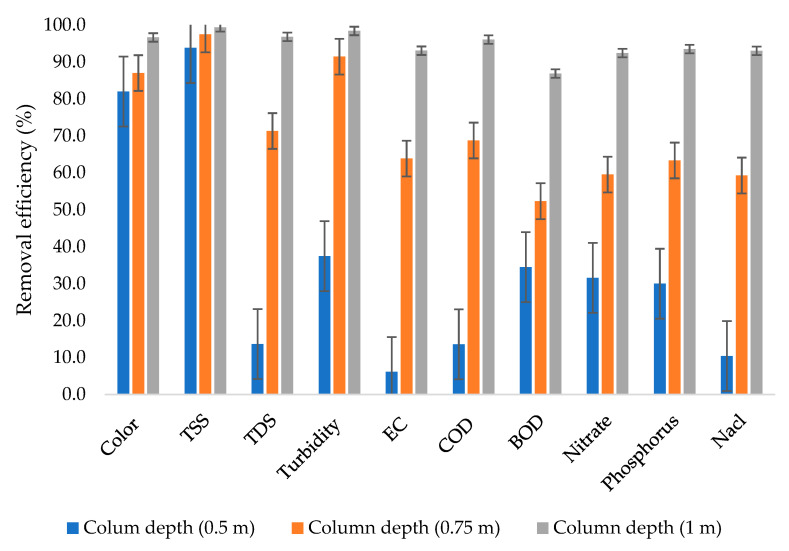
Removal efficiencies from different column depths.

Figure 3 shows that there was very high variability in terms of removal efficiency when the wastewater was subjected to a 0.5 m column depth with removal efficiencies ranging from 6.1% to 93.8%, with the lowest removal efficiency observed from EC. The phenomenon is probably due to the fact that the column depth was not sufficient enough to remove the majority of the dissolved solids, including phosphorus. In the literature, some other studies, such as the one conducted by Urminská et al. [47], observed that natural zeolite faces a significant challenge in the removal of phosphorus. 

From a 0.75 m column depth, the majority of the dissolved solids were removed, reaching up to 71.3% removal efficiency. The results in this study are in agreement with the results obtained from the study conducted by Yogafanny et al. [48], in which groundwater was treated using natural zeolite filter material with a grain size of 0.1 cm, resulting in a removal efficiency of 79.76% being achieved for TDS. Despite the low removal efficiency for TDS from a 0.5 m column depth, the column still showed impressive performance for some other parameters, such as TSS. 

In general, the 0.75 m column depth achieved removal efficiencies ranging from 52.3% to 97.5%, whereas the 1 m column depth achieved removal efficiencies ranging from 86.9% to 99.4%. The highest removal efficiency was achieved from the combination of TSS and the 1 m column depth. The results in this study are in agreement with the results obtained from the study conducted by El beid et al. [49], which investigated the performance of the filtration process for the treatment of pig slurry by using marine sands, silty loam soils, fly ash, and zeolite; in the study, a column of 0.5 m of zeolite was used to achieve removal efficiencies of up to 99.9% for EC, TSS, COD, and BOD.

Moreover, in the literature, similar results were observed when wastewater from the primary sedimentation basin was treated by integrating natural zeolite and lime [50], where up to 99% removal efficiency was achieved from TSS.

### 2.7. The Effect of pH on the Performance of the Filter Material

**Figure 4:**
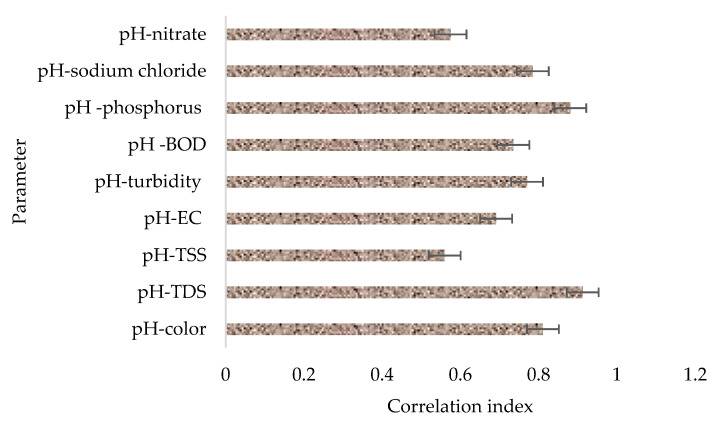
The potential influence of pH on the selected parameters based on correlation indices.

Figure 4 provides a summary of the potential influence of initial pH in the general performance of the filter material based on correlation matrices or indices. It is also worth remembering that the pH in the raw wastewater ranged from 7.5 to 12.3. From the analysis results, it was observed that there was a strong correlation between the initial pH and the concentration of contaminants remaining in the treated effluent. It was specifically observed that the treatment system performed better when the initial pH in the samples was close to neutral (7.5) while decreasing a bit when the wastewater was highly alkaline (12.3). The results in this study suggest that the pH of the contact wastewater can negatively impact the adsorption capacity of zeolite, especially at high pH values, with the potential of a similar effect at relatively low pH values. The results are in agreement with the study conducted by Margeta et al. [51], in which the authors reported that zeolite showed the best performance on the removal of the studied pollutants at a pH close to that of natural waters.

Also, in a study conducted by Cozmuta et al. [52] that investigated the potential influence of pH on the adsorption of lead using zeolite, it was observed that when the pH of the solution decreased from 4 to 1, the adsorption capacity of the filter material also decreased to about 72.72%.

### 2.8. Adsorption Capacity of the Filter Material

**Figure 5:**
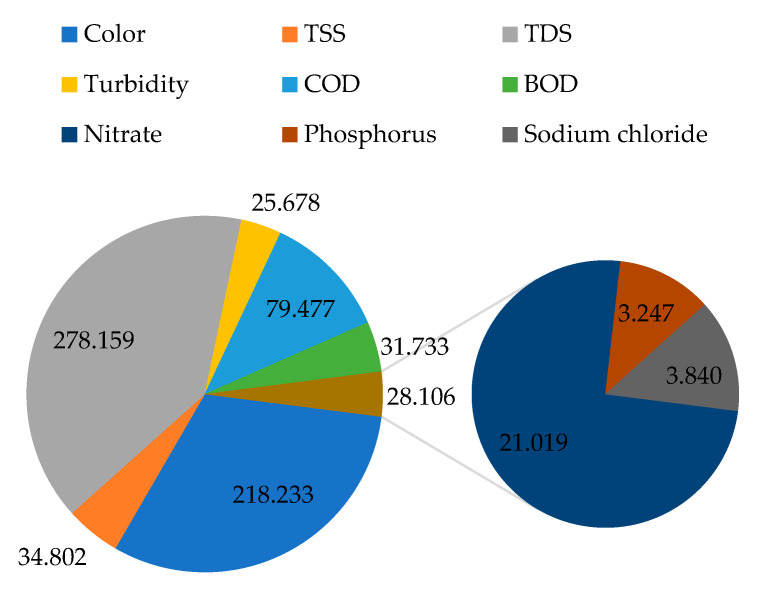
The estimated adsorption capacity of the filter material (mg/g).

Figure 5 presents a summary of the computed adsorption capacity of the filter material for different parameters in mg of a pollutant per g of filter material from a 1 m column depth. From Figure 5, it can be observed that the filter material was able to capture about 218.233 mg of color per g of the filter material. In the literature, some other filter materials, such as activated carbon, have also been investigated for their potential adsorption capacity in terms of color; for instance, in a study conducted by El maguana et al. [53] on the removal of dyes using activated carbon, it was observed that the material had an adsorption capacity of 336.12 mg/g at a pH of 7, which is slightly higher than the value obtained with natural zeolite in this study at pH values between 7.5 and 12.3. 

Also, 34.802 mg of TSS was captured per g of filter material. Zeolites are characterized by a relatively large surface area and porosity that allow proper capturing of suspended particles in wastewater to the extent applied to soils to increase their sorption properties [54]. 

From TDS, 278.159 mg was captured per g of the filter material, while from turbidity, 25.678 mg was captured. In summary, 79.477, 31.733, 21.019, 3.247, and 3.840 were captured for COD, BOD, nitrate, phosphorus, and sodium chloride, respectively. The observed low adsorption capacities for phosphorus and sodium chloride in comparison to the other investigated water quality parameters are closely linked to the fact that the concentrations of phosphorus and sodium chloride were already relatively low in the raw wastewater with respect to the amount of zeolite material used. In that matter, for phosphorus-rich wastewater, the adsorption capacity of the zeolite filter material can significantly improve, especially with larger column depths.

In the literature, up to 96.74 mg/g adsorption capacity for COD was achieved when zeolite was mixed with activated carbon [55], which is close to the adsorption capacity value obtained in this study with natural zeolite alone.

## 3. Materials and Methods

### 3.1. Case Study Description, Analytical Methods, and Wastewater Characteristics

The textile raw wastewater samples used in this study were collected from the Nida Textile Mills located in Dar es Salaam City, Tanzania, approximately 10.9 km from Julius Nyerere International Airport (Latitude: 6°49′8.50″ S, Longitude: 39°17′2.57″ E). The mills were established in 2002 as a company with an integrated manufacturing process that includes the processing of cotton at source to yarn and finished textiles.

**Figure 6:**
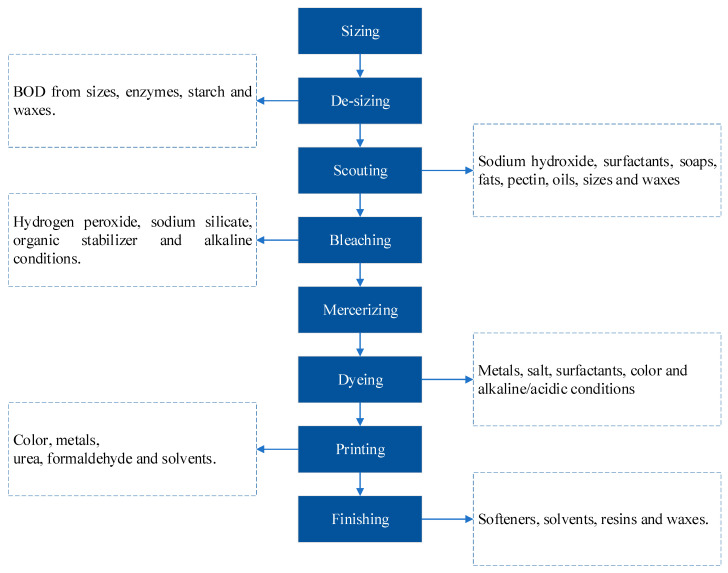
General textile industry production processes.

In general, any textile industry encompasses a significant number and variety of processes that are important in adding value to fiber. These processes may range from yarn making through garment stitching, fabric embossing, and composite production. Figure 6 provides a summary of the general production processes of a textile industry [56].

Generally, the wastewater samples were collected using a discrete sampling approach using 10 L and 20 L plastic buckets that were properly rinsed before being subjected to the samples. To make sure that data quality remained under control, replicate samples were also collected and analyzed. All samples were preserved at 4 °C before treatment and analysis. The team attempted to treat and analyze all the samples collected within the same day of collection.

Moreover, different scientific procedures, kits, and reagents were used to assess the parameters of interest in the water samples. Physical parameters in the raw wastewater, such as pH, temperature, dissolved oxygen (DO), electrical conductivity (EC), and total dissolved solids (TDS), were measured on-site using the Hanna Multiparameter-HI 9829 (Hanna Instruments, George Washington Hwy, Chesapeake, VA, USA), while the turbidity in the samples was determined using Hanna Turbidometer -HI 93703 (Hanna Instruments, George Washington Hwy, Chesapeake, VA, USA). Color, chemical oxygen demand (COD), nitrate, phosphorus, ammonia, and total suspended solids (TSS) were determined using a DR2800 spectrophotometer (Hach Company, Lindbergh Drive Loveland, CO, USA). Generally, all the studied samples were analysed following the recommendations in the APHA Standard Methods for the Examination of Water and Wastewater [57].

Table 6 presents a summary of the raw wastewater characteristics in terms of minimum (Min) and maximum (Max) median, arithmetic mean (AM), and standard deviation (STD).

### 3.2. Experimental Setup

**Figure 7:**
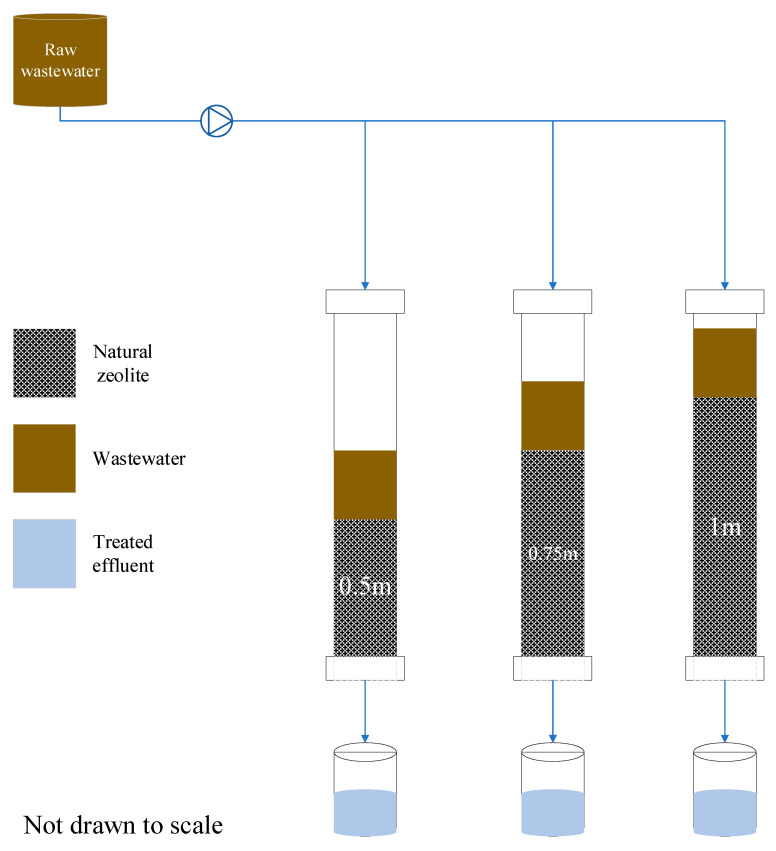
Experimental setup.

The experimental setup consisted of three fixed-bed zeolite columns with 0.5 m, 0.75 m, and 1 m depths. The columns were divided into different depths to investigate the influence of column depth in the textile wastewater treatment efficiency using zeolites (Figure 7). The column containers were made of Polyvinyl chloride (PVC) material, approximately 5.08 cm in diameter each. All three columns were packed with natural zeolite adsorbents (clinoptilolite) composed of a microporous arrangement of silica and alumina tetrahedra, with an average particle size of 1.5 mm (FM Stock and Supplies, Kenmare, Gauteng, South Africa).

It was also important to take into account the equal distribution of flow in the columns and, to achieve this, the top surfaces of the columns were covered by perforated plates with evenly distributed holes. The system used a 120 L storage drum to continually feed the columns at a controlled rate of (0.0035 L/s). The wet-packing approach of the porous medium was used to minimize layering and air entrapment inside the filing. All three zeolite columns were mounted vertically, and glass wool was used at the bottom of the column, acting as a supporting material of the adsorbent bed. After packing the column, deionized water was passed through it for some time, followed by the introduction of the feed water. The filtrate samples were collected at a regular time interval (every 2 h on average, when the collection jars were almost full). To avoid data errors resulting from an exhausted (saturated) media treating the highly polluted wastewater and only capturing the effect of column depth on the removal efficiency, the media had to be changed after every five days.

### 3.3. Statistical Methods

#### 3.3.1. Relationship among Parameters

The study involved the determination of relationships among the studied parameters. To achieve this, correlation matrices were developed as one approach used to analyze results from the experiments. In that matter, correlation matrices were developed to evaluate the strength of the relationship among the studied parameters. In general, a high correlation indicated that two or more variables had a strong relationship with each other, while a weak correlation meant that the variables were hardly related [58]. Table 7 provides a summary of the interpretation of the correlation indices used in this study.

#### 3.3.2. Removal Efficiency Calculation

After the analysis of raw wastewater (before treatment) and purified water, the results were subjected to the determination of removal efficiencies, presented as percentages for better visualization of the performance from the studied treatment approaches with respect to the studied electrode materials. The approach used for the treatment efficiency computations is summarized in Equation (3).

(3) $$T_{e}\left(\%\right)=\frac{C_{b}-C_{a}}{C_{b}}\times 100$$

where *T_e_* is treatment efficiency, *C_b_* is the initial concentration from raw wastewater, and *C_a_* is the concentration of a parameter. 

#### 3.3.3. Data Distribution Analysis

Box and whisker plots were also developed and used for water quality data distribution analysis. They visually showed the distribution of numerical data and its skewness by displaying the data quartiles (percentiles) and averages. From the perspective of statistical analysis, box plots show the five-number summary of a set of data, including the minimum score, first (lower) quartile, median, third (upper) quartile, and maximum score [59]. 

#### 3.3.4. Estimation of Filter Adsorption Capacity

The adsorption capacity (*q*) (in mg per gram unit of zeolite weight) of the filter material was estimated based on the initial concentration of a parameter in the raw wastewater (*C_r_*) and the concentration of the particular parameter in a treated effluent (*C_t_*). Some other parameters, such as the weight of the material used, and the volume of the wastewater subjected to the treatment system, were also used. In summary, adsorption capacity (also known as the efficiency of adsorption) is a ratio of the amount of adsorbate adsorbed by a filter material to the amount of adsorbent material used for the adsorption process (Equation (4)).

(4) $$q=\frac{\left[\left(C_{r}-C_{t}\right)\times V\right]}{m}$$

## 4. Conclusions

In this study, the potential effect of a column depth on the treatment of textile wastewater using natural zeolite was investigated. The results indicated that the filtration process using natural zeolite was highly effective in removing the majority of the pollutants in the wastewater. However, the removal efficiency was affected by the column depth, and an increase in the column depth also increased the removal efficiency of the treatment systems. The highest removal efficiency (99.4%) was achieved from the combination of TSS and a 1 m column depth. It was also observed that the 0.5 m column depth somehow struggled in removing some of the water quality parameters, such as EC, with a removal efficiency of only 6.1%. The phenomenon probably occurred because the depth was not sufficient enough to remove the majority of the dissolved solids. However, the column depth showed impressive performance on the removal of certain parameters, such as color and TSS. The results from this study further revealed that natural zeolite was highly effective in purifying textile wastewater; however, the optimum depth to achieve a desirable performance should always be investigated before fixing the final (working) treatment system. 

## Figures and Tables

**Table 1:** Relationship among color, TSS, TDS, DO, and turbidity in the raw wastewater.

	Color	TSS	TDS	DO	Turbidity
Color	1				
TSS	0.94	1			
TDS	0.73	0.59	1		
DO	−0.71	−0.47	−0.66	1	
Turbidity	0.99	0.95	0.69	−0.66	1

**Table 2:** Relationship among DO, TDS, EC, COD, and BOD in the raw wastewater.

	DO	TDS	EC	COD	BOD
DO	1				
TDS	−0.66	1			
EC	−0.90	0.86	1		
COD	−0.83	0.56	0.88	1	
BOD	−0.92	0.88	0.99	0.84	1

**Table 3:** Water quality characteristics from the samples collected from 0.5 m column depth.

Parameter	Max	Min	Median	Mean	STD
Color	505	185	262.5	303.75	123.46
TSS	72	19	42	43.6	16.86
TDS	7860	4280	6846	6458	1417.36
DO	3	1.6	2.55	2.425	0.51
Turbidity	172.5	12.2	114	103.175	59.09
EC	8862	5286	6849	6961.5	1285.20
COD	4000	330	1522	1843.5	1339.38
BOD	500	100	180.5	240.25	158.46
Nitrate	47	4.6	31.5	31.22	15.22
Phosphorus	7.4	0.6	4	4	3.40
Sodium chloride	9.7	7.08	9.05	8.72	0.99

**Table 4:** Water quality characteristics from the samples collected from 0.75 m column depth.

Parameter	Max	Min	Median	Mean	STD
Color	287.5	110	221.5	219.25	60.50
TSS	25	11	16	17.83	5.37
TDS	2680	1220	2395	2146.50	583.45
DO	8.9	4.3	6	6.38	1.90
Turbidity	24	5	11.75	14.15	7.00
EC	3160	2220	2655	2682.67	291.84
COD	2250	113	350	667.17	739.59
BOD	250	120	177.5	174.83	44.54
Nitrate	48	1	14.75	18.47	14.60
Phosphorus	2.4	0.2	1.5	1.47	0.69
Sodium chloride	5	2.9	4.14	3.96	0.81

**Table 5:** Water quality characteristics from the samples collected from a 1 m column depth.

Parameter	Max	Min	Median	Mean	STD
Color	90	10	85	56.90	37.52
TSS	7	1	5	4.50	1.80
TDS	469	89	205.5	241.00	136.77
DO	18.9	8.2	10.6	12.55	4.70
Turbidity	5	0.4	2.75	2.65	1.60
EC	884	160	576	516.00	254.85
COD	250	13	65	83.83	79.48
BOD	80	20	45	48.17	23.02
Nitrate	8	1	1.9	3.47	2.91
Phosphorus	0.6	0	0.2	0.26	0.22
Sodium chloride	2.2	0	0.45	0.68	0.75

**Table 6:** The general characterization of the raw wastewater characteristics (number of samples = 12).

Parameter	Max	Min	Median	Mean	STD
pH	12.3	7.5	10.05	9.917	1.61
Color	4090	231	1598.75	1685.92	1211.78
TSS	2435	131	294.5	702.17	813.94
TDS	10,160	2680	8575	7479.83	2547.35
DO	7.7	1.2	1.6	2.88	2.34
Turbidity	450	9.4	148.5	164.98	138.2
EC	13,460	534.4	9840	8249.067	4295.098
COD	3750	480	2151.5	2133.83	946.68
BOD	550	125	425	366.67	152.53
Nitrate	170	1	21.9	45.63	57.69
Phosphorus	7.8	0.2	2.2	2.8	2.4
Sodium chloride	13	2.9	10.55	9.7	3.34

EC in μS/cm, color in degrees, and all other parameters in mg/L.

**Table 7:** Interpretation of the correlation coefficients.

Range of Correlation Coefficient	Strength of Relationship
0–0.29	Weak
0.3–0.49	Moderate
0.5–0.69	Strong
0.7–1	Very strong

## Data Availability

Not applicable.
